# First record of *Caligusdussumieri* Rangnekar, 1957 (Copepoda, Siphonostomatoida, Caligidae) from Malaysia, with notes on caligids found from Malaysia and on host-specificity of caligids on lutjanid fishes

**DOI:** 10.3897/BDJ.12.e116598

**Published:** 2024-02-20

**Authors:** Norshida Ismail, Yusuke Nishida, Susumu Ohtsuka, Geoff Boxshall, James P. Bernot

**Affiliations:** 1 Faculty of Bioresources and Food Industry, Universiti Sultan Zainal Abidin, 22200, Besut, Terengganu, Malaysia Faculty of Bioresources and Food Industry, Universiti Sultan Zainal Abidin, 22200, Besut Terengganu Malaysia; 2 Graduate School of Integrated Sciences for Life, Hiroshima University, 1-4-4 Kagamiyama, Higashi-Hiroshima, 739-8528, Hiroshima, Japan Graduate School of Integrated Sciences for Life, Hiroshima University, 1-4-4 Kagamiyama, Higashi-Hiroshima, 739-8528 Hiroshima Japan; 3 Blue Innovation Division, Seto Inland Sea Carbon Neutral Research Center, 5-8-1 Minato-machi, Takehara, 725-0024, Hiroshima, Japan Blue Innovation Division, Seto Inland Sea Carbon Neutral Research Center, 5-8-1 Minato-machi, Takehara, 725-0024 Hiroshima Japan; 4 Department of Life Sciences, The Natural History Museum, Cromwell Road, London SW7 5BD, UK., London, United Kingdom Department of Life Sciences, The Natural History Museum, Cromwell Road, London SW7 5BD, UK. London United Kingdom; 5 Department of Invertebrate Zoology, National Museum of Natural History, Smithsonian Institution, 20560, Washington DC, United States of America Department of Invertebrate Zoology, National Museum of Natural History, Smithsonian Institution, 20560 Washington DC United States of America; 6 Department of Ecology and Evolutionary Biology, University of Connecticut, Storrs, 06269, Connecticut, United States of America Department of Ecology and Evolutionary Biology, University of Connecticut, Storrs, 06269 Connecticut United States of America

**Keywords:** Caligidae, Lutjanidae, *bonito*-species group, sea lice, identification key, geographic distribution, host list

## Abstract

**Background:**

In total, 14 species of *Caligus* have been reported from Malaysia. Amongst them, four species are reported from lutjanid fishes.

**New information:**

*Caligusdussumieri* Rangnekar, 1957 is reported from Malabar snapper, *Lutjanusmalabaricus*, purchased from a local wet market in Terengganu, Peninsular Malaysia. This is the first record of this species in Malaysia and it is only the second species assigned to the *bonito*-group of the genus *Caligus* to be reported from Malaysia. A key to species of the *bonito*-group is presented herein. The list of caligids infecting lutjanid fishes and the geographical distributions plus the known hosts of members of the *bonito*-group of *Caligus* are discussed.

## Introduction

Parasitic copepods of the genus *Caligus* Müller, 1785 (Siphonostomatoida, Caligidae) are well known as pests that cause serious economic losses in the marine aquaculture and fisheries industries globally (Ho and Lin 2004, Johnson et al. 2004, Nagasawa 2013, Arriagada et al. 2019, Hemmingsen et al. 2020). Heavy infestation by caligids, especially in fish kept in captivity, often results in secondary infection and disease outbreaks (Johnson et al. 2004). Therefore, the taxonomy, ecology and physiology of sea lice are particularly important and intensively studied.

To date, 269 valid species of *Caligus* have been described from a wide diversity of fish hosts (Walter and Boxshall 2023). Despite the exceptional diversity of aquatic organisms in Malaysia with approximately 1,955 fish species recorded in FishBase (Froese and Pauly 2023), only 27 identified species of parasitic copepods have been reported (Arthur and Shariff 2015, Ohtsuka et al. 2020a, Yahaya et al. 2022, Venmathi Maran et al. 2022). Amongst these, 16 species belong to the family Caligidae, namely: *Caliguschiastos* Lin and Ho, 2003, *C.clemensi* Parker and Margolis, 1964, *C.eleutheronemi* Shen, 1957, *C.epidemicus* Hewitt, 1971, *C.epinepheli* Yamaguti, 1936, *C.hirsutus* Bassett-Smith, 1898 (as *Parapetalushirsutus* Basset-Smith, 1898), *C.kanagurta* Pillai, 1961, *C.laticaudus* Shiino, 1960, *C.longipedis* Basset-Smith, 1898, *C.minimus* Otto, 1821, *C.malabaricus* Pillai, 1961, *C.punctatus* Shiino, 1955, *C.torpedinis* Heller, 1865 (as *C.rotundigenitalis* Yu, 1933), *C.stromatei* Krøyer, 1863 (as *C.multispinosus* Shen, 1957), *Hermiliuslongicornis* Basset-Smith, 1898 and *H.pyreventris* Heller, 1865 (Leong 1984, Leong 1985, Venmathi Maran et al. 2009, Khoa et al. 2019, Yahaya et al. 2022).

Seven species groups have been established within the genus *Caligus*: the *bonito*-, *confusus*-, *diaphanus*-, *macarovi*-, *productus*-, *pseudrorhombi*- and *undulatus*-groups (Boxshall 2018, Ohtsuka and Boxshall 2019, Ohtsuka et al. 2020b). The *bonito*-group can be recognised on the basis of the following features: well-developed inner setae on the distal exopodal segment of leg 1 and the presence of a row of robust denticles along the outer margin of the second endopodal segment of leg 2.

The fish genus *Lutjanus* Bloch, 1790 (Lutjanidae) plays an important role in fisheries and aquaculture in Malaysia. The lutjanid catch contributed about 5.3% of the overall marine fish catch in 2021 (DOFM 2021). Bakar et al. (2018) identified 17 species of *Lutjanus* from Malaysia. Amongst them, *Lutjanusmalabaricus* (Bloch and Schneider, 1801), commonly known as Malabar snapper, is one of the economically important species as they are recorded in 10 fish landing facilities across Malaysia: in Kedah, Penang, Perak, Selangor, Johor, Terengganu, Kelantan and Sabah (DOFM 2021). To date, only one species of parasitic copepod (*C.malabaricus*) has been described, associated with *L.malabaricus* from Malaysia (Leong 1984).

We have conducted taxonomic studies on metazoan parasites on commercially important fish obtained in the fish markets in Terengganu since 2019 (Ohtsuka et al. 2020b, Nitta et al. 2022) and, in this paper, we report a new locality record for *Caligusdussumieri* Rangnekar, 1957 from Malabar snapper, *Lutjanusmalabaricus*. This species is re-described herein and we present notes on *Caligus* species parasitic on *Lutjanus* around the world and on the host-specificity of members of the *bonito*-group.

## Materials and methods

A single female specimen of *Caligusdussumieri* was found on the gills of *L.malabaricus* purchased from the fish landing facility of the Fisheries Development Authority of Malaysia (LKIM) Complex Pulau Kambing, located in Kuala Terengganu, Terengganu State, Malaysia on 19 October 2019. The fish host was purchased dead and brought back to be examined for parasites in the Aquatic Laboratory, Faculty of Bioresources and Food Industry, University Sultan Zainal Abidin Besut Campus. The body surface, head and gills of the host were examined for parasites. Parasite specimens were fixed in 70% ethanol until identification. The copepod specimen was immersed in lactophenol and examined using the Humes and Gooding (1964) slide method, on a differential interference microscope (BX-53, Olympus Co., Ltd.), equipped with a drawing tube. The body length was measured from the frontal margin of the cephalothorax to the posterior margin of the caudal ramus excluding the caudal setae. The specimen is deposited at the South China Sea Repository and Reference Center, Universiti Malaysia Terengganu, Malaysia (UMTCrus 1645). A second specimen of *C.dussumieri* from the Natural History Museum, London (Reg. No. NHMUK 2022.189–197), fixed in 70% ethanol and collected from *L.johnii* from the Ord River, Camden Sound, Western Australia on 11 September 2013, was also examined. It was cleared in lactophenol on a glass cavity slide and examined with light microscopy using a Leitz Diaplan microscope, equipped with differential interference contrast and with confocal laser scanning microscopy (CLSM) as detailed below. Terminology of adults and developmental stages follows Huys and Boxshall (1991), Ho and Lin (2004) and Piasecki et al. (2023).

### Confocal Laser Scanning Microscopy

One female of *Caligusdussumieri* (NHMUK 2022.189–197) was examined using CLSM. The specimen was stained overnight in a saturated solution of Congo Red in 100% ethanol, then rinsed in distilled water until no Congo Red could be seen diffusing and prepared as a temporary mount in a 50% solution of glycerine and distilled water on a glass slide under a coverslip. The specimen was examined using a Leica TCS SP5, equipped with a Leica DM5000 B upright microscope and the Leica Application Suite Advanced Fluorescence software LAS AF 2.2.1. (Leica, Wetzlar, Germany). We used a 561 nm excitation wavelength from a DPSS 10 mW 561 nm laser set at 80% power and collected the emitted fluorescence in two channels: 570–630 nm artificially coloured green and 630–715 nm artificially coloured red. A series of image stacks were collected and the final images were obtained by maximum projection of the overlaid channels using the same Leica software. For the full-body dorsal and ventral images, multiple fields of view were combined using Adobe Photoshop v.25.1.

## Taxon treatments

### Caligus dussumieri

Rangnekar, 1957

A5E4758E-FFD4-5223-8596-82A71425C8C2

*Sinocaligusdussumieri* (Rangnekar, 1957)
*Pseudopetalusdussumieri* (Rangnekar, 1957)
*Caligusrivulatus* (Pilla, Vankara and Chikkam, 2012)

#### Materials

- **Type status:** Other material. **Occurrence:** occurrenceID: A71C4FDE-7825-5C4B-9023-C44E1AC10847; **Taxon:** taxonID: 349612 (urn:lsid:marinespecies.org:taxname:349612); scientificName: *Caligusdussumieri* Rangnekar, 1957; parentNameUsage: *Caligus* Müller O.F., 1785; originalNameUsage: *Caligusdussumieri* Rangnekar, 1957; kingdom: Animalia; phylum: Arthropoda; class: Copepoda; order: Siphonostomatoida; family: Caligidae; genus: Caligus; specificEpithet: dussumieri; scientificNameAuthorship: Rangnekar, 1957; taxonomicStatus: Accepted; **Location:** higherGeography: South East Asia; continent: Asia; waterBody: South China Sea; country: Malaysia; stateProvince: Terengganu; county: Kuala Terengganu; verbatimLocality: LKIM Pulau Kambing; verbatimCoordinates: 5°19'N 103°7' 43.7"E; verbatimLatitude: 5.321971; verbatimLongitude: 103.1288; **Identification:** identificationID: urn:lsid:marinespecies.org:taxname:349612; **Event:** samplingProtocol: Gill wash; year: 2019; month: 10; day: 19
- **Type status:** Other material. **Occurrence:** occurrenceID: ADE72616-49DB-57BB-89BA-3948143646FA; **Taxon:** taxonID: 349612 (urn:lsid:marinespecies.org:taxname:349612); scientificName: *Caligusdussumieri* Rangnekar, 1957; parentNameUsage: *Caligus* Müller O.F., 1785; originalNameUsage: *Caligusdussumieri* Rangnekar, 1957; kingdom: Animalia; phylum: Arthropoda; class: Copepoda; order: Siphonostomatoida; family: Caligidae; genus: Caligus; specificEpithet: dussumieri; scientificNameAuthorship: Rangnekar, 1957; taxonomicStatus: Accepted; **Location:** higherGeography: Australia; continent: Australia; country: Australia; county: Camden Sound; locality: Western Australia; verbatimCoordinates: 16° 11.52’S 124° 32.52’E; **Identification:** identificationID: urn:lsid:marinespecies.org:taxname:349612; **Event:** year: 2013; month: 9; day: 11; **Record Level:** institutionCode: NHMUK; collectionCode: Reg. Nos. 2022.189-197

#### Description

Fig. 1

**Figure 1.**
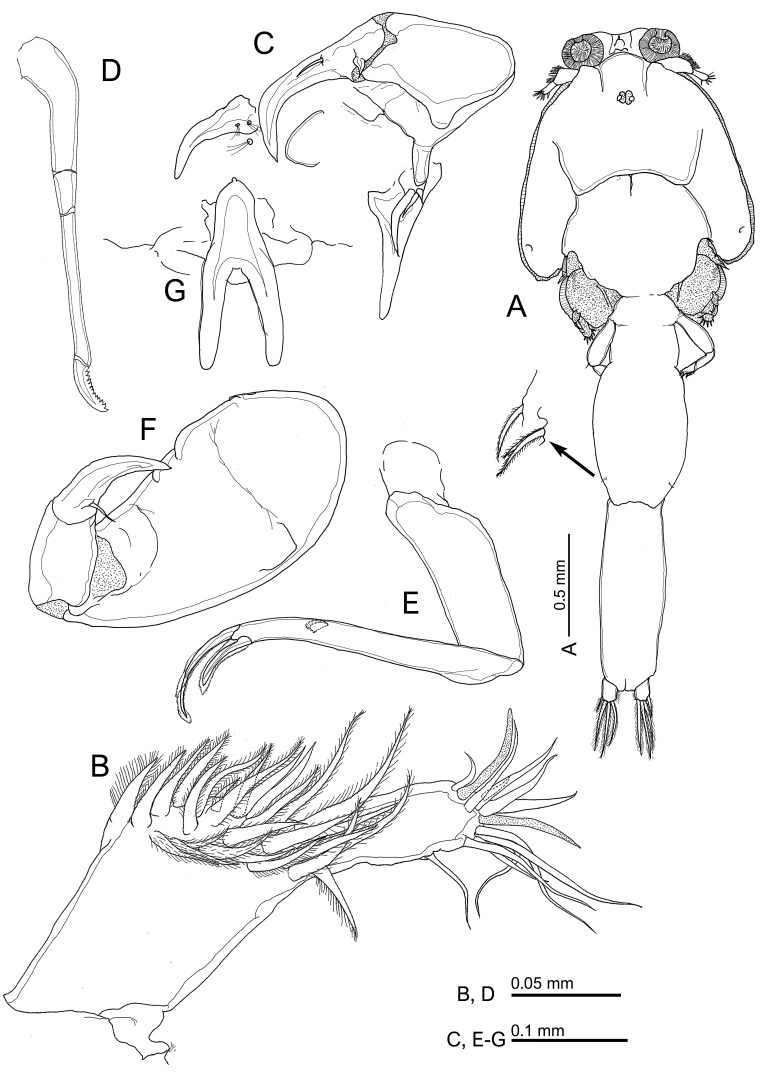
*Caligusdussumieri* Rangnekar, 1957, adult female. **A** Habitus, dorsal view; **B** Antennule; **C** Antenna; **D** Mandible; **E** Maxilla; **F** Maxilliped; **G** Sternal furca.

**Figure 2.**
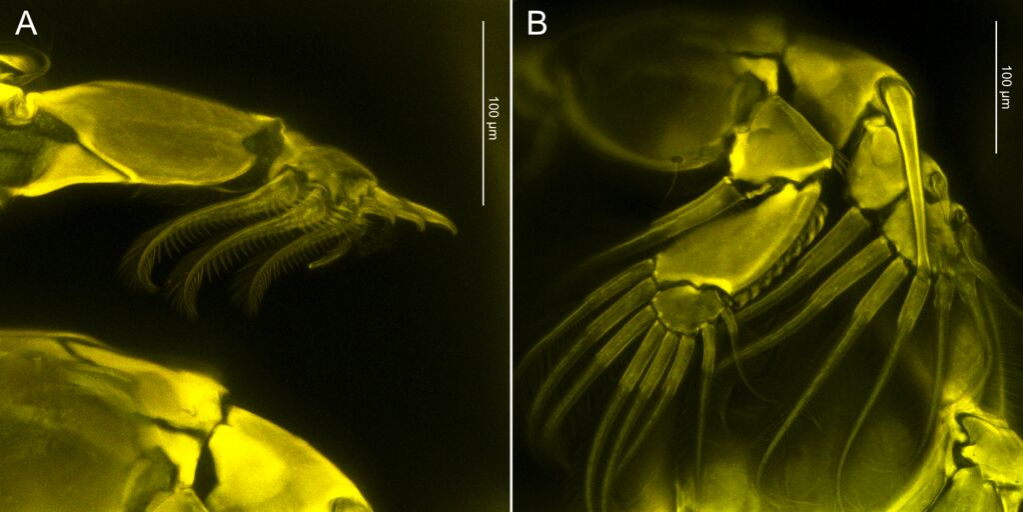
CLSM images of *Caligusdussumieri* Rangnekar, 1957. **A** Exopod of leg 1, showing spinular ornamentation on plumose setae along posterior margin; **B** Rami of leg 2 showing ornamentation on outer margin of endopod and spatulate spine on first exopodal segment.

**Female.** Body (Fig. 1A) 3.24 mm long, excluding setae on caudal ramus. Dorsal cephalothoracic shield subcircular, slightly longer than wide. 1.31 x 1.12 mm, excluding hyaline membrane along lateral margins. Shield tapering anteriorly; with large, conspicuous lunules. Fourth pediger wider than long, incompletely fused to genital complex. Genital complex about 1.96 times longer than wide with neck-like narrow part anteriorly and barrel-shaped posterior region. Leg 5 (Fig. 1A) represented by 1 plumose, outer protopodal seta and 2 plumose setae on exopodal papilla located on posterolateral margin of genital complex. Abdomen 1-segmented, about 2.8 times longer than wide. Caudal ramus (Fig. 2C) oval, 0.10 x 0.07 mm, expanded inwards distally; armed with 3 short and 3 long plumose setae and ornamented with row of setules along distomedial margin.

**Figure 3.**
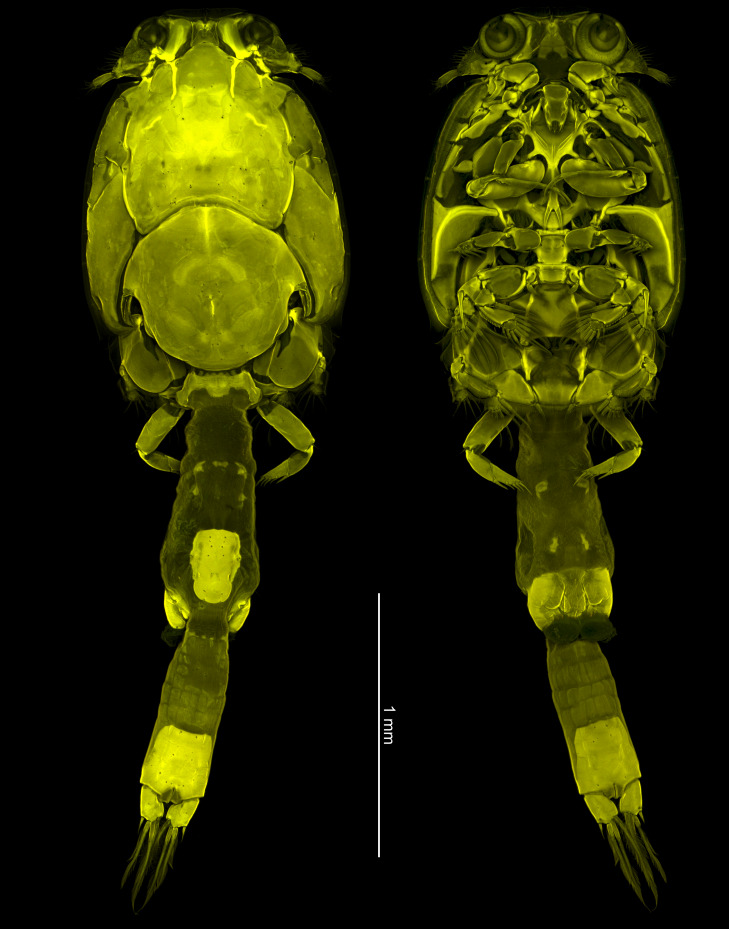
CLSM images of *Caligusdussumieri* Rangnekar, 1957. **A** dorsal view; **B** ventral view.

Australian female observed using CLSM (Fig. 3A, B) showing differential degree of staining over body surface. Dorsal cephalothoracic shield staining moderately and evenly, with heavier uptake of stain marking sutures anteriorly near origin of antennules, in area near eyes and along lateral edges of central thoracic zone of shield. Ventral curved rib marking boundary of cephalosome and incorporated first pediger strongly stained. Fourth pediger and fourth leg staining evenly. Genital complex and abdomen displaying marked differences in intensity of staining. Staining intense in small ovoid patch on dorsal surface of genital complex towards the posterior end, ventrally around the genital apertures and in the posterior part of the abdomen. Conversely, staining very light in other areas of genital complex and anterior part of abdomen.

Antennule (Fig. 1B) 2-segmented; proximal segment carrying 27 plumose setae on anterodistal surface, 25 setose plus 2 naked (on dorsal side) setae; distal segment short with 1 subterminal seta on posterior margin and 11 setae plus 2 aesthetascs around apex. Antenna (Fig. 1C) 3-segmented; proximal segment smallest, with short blunt process on posteromedial corner; middle segment subrectangular, unarmed; distal segment forming long, curved claw armed with stout proximal seta and simple middle seta. Postantennal process (Fig. 1C) shorter than distal segment, tine curved inwards with blunt tip, ornamented with 2 multisensillate papillae on basal region and another multisensillate papillae present on sternum adjacent to base of process. Additional lobate process present between postantennal process and base of antenna. Mandible (Fig. 1D) stylet-like, with 4 sections, bearing 12 teeth on medial margin of distal blade. Maxillule (Fig. 1C) comprising large stout dentiform process and basal papilla with 3 setae (1 short, 1 medium and 1 long). Maxilla (Fig. 1E) 2-segmented; lacertus large and unarmed; slender distal segment (brachium) bearing rounded membranous subapical flabellum on anterior margin and terminating in 2 subequal claw-like elements (calamus and canna). Calamus longer than canna, ornamented with strips of serrated membrane arranged obliquely around surface; canna ornamented with strips of serrated membrane bilaterally. Maxilliped (Fig. 1F) 3-segmented; proximal segment (corpus) largest, unarmed, 2 unequal processes along myxal area, longer than next 2 segments combined (subchela); middle segment unarmed; distal claw longer than shaft, sharply pointed with long seta at base of claw. Sternal furca (Fig. 1G) subrectangular, box with tiny rounded outgrowth anteriorly, tines blunt and weakly divergent.

**Figure 4.**
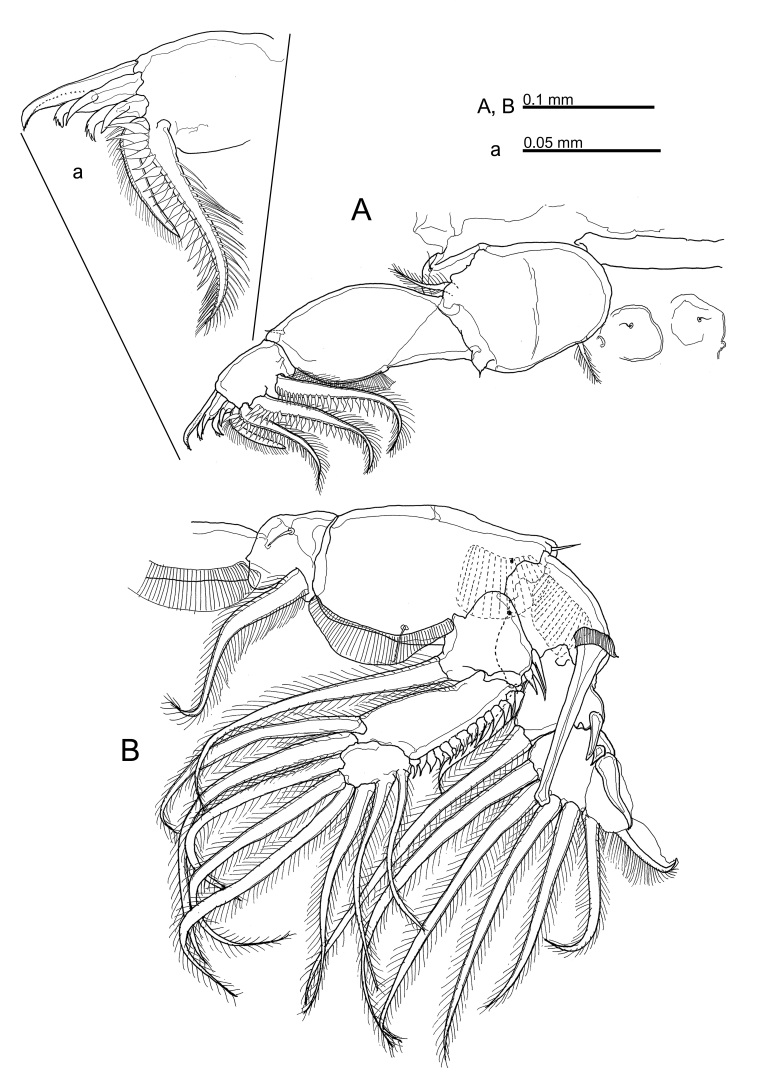
*Caligusdussumieri* Rangnekar, 1957 adult female. **A** Leg 1, **a** Four terminal elements of Leg 1; **B** Leg 2.

**Figure 5.**
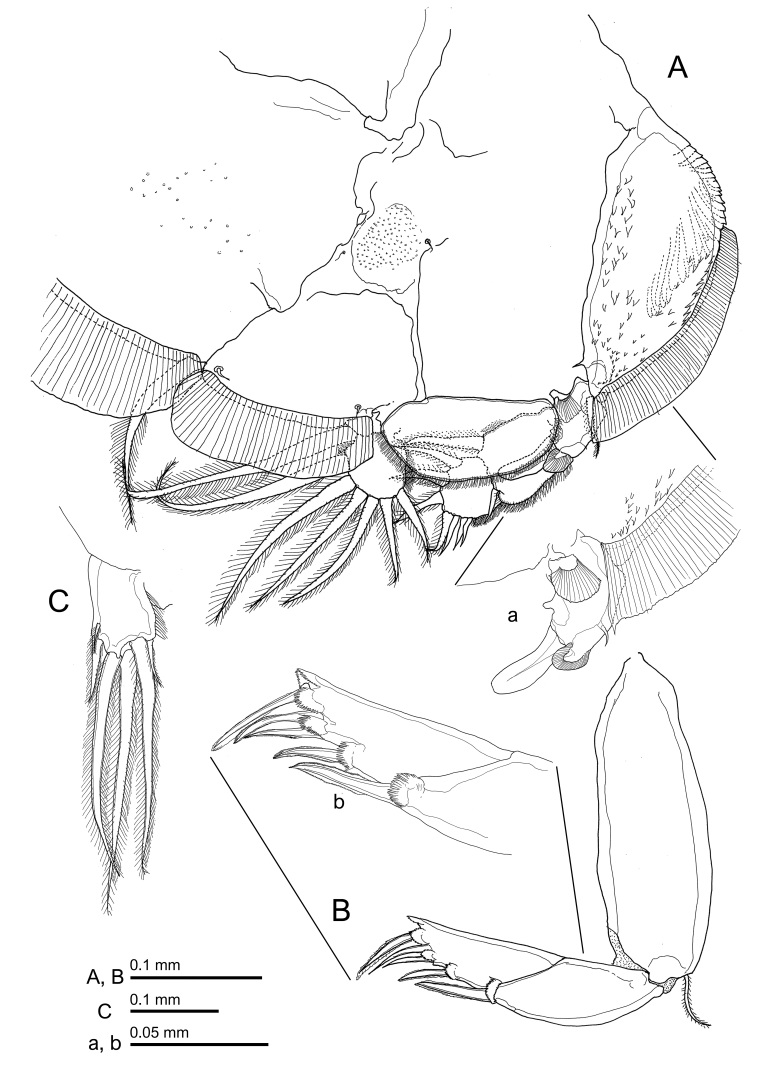
*Caligusdussumieri* Rangnekar, 1957, adult female. **A** Leg 3, **a** Proximal segment of leg 3 exopod; **B** Leg 4, **b** Distal elements of Leg 4 second segment.

Leg 1 (Fig. 4A, Fig. 2A) with 2 segmented protopod bearing 1 inner and 1 outer small plumose seta plus bifid setule on outer margin. Intercoxal sclerite slender, with 2 papillae each bearing a setule present on sternum posterior to sclerite. Vestigial endopod reduced to tiny, pointed process located near base of exopod. Exopod 2-segmented, first segment with row of fine setules along inner margin and spiniform seta at outer distal corner; second segment with 3 large plumose setae ornamented along outer margin with stout spinules at base becoming finer towards tip and with short plumosities along inner margin; 4 terminal elements, spine 1 simple, spines 2 and 3 each with accessory process (Fig. 4a), seta 4 longest, stout and pinnate on only one side. Leg 2 (Fig. 4B, Fig. 2B) with intercoxal sclerite ornamented with marginal membrane along posterior margin; coxa with large plumose seta at posterior corner and minute setule on anterior surface; basis ornamented with marginal membrane on both inner and medial margins, bearing minute seta at outer distal corner plus setule near mid-point of inner margin. Endopod 3-segmented: first segment armed with inner plumose seta and ornamented with large denticles at outer distal corner; second segment with 2 inner distal setae plus row of robust denticles along outer margin; third segment with 6 plumose setae. Exopod 3-segmented: first segment with reflexed membrane dorsally and with pecten at base of long, stout, outer spine with spatulate tip extending obliquely across surface of second and third segments; second segment with relatively short outer spine, third segment armed with 1 small and 1 medium outer spine (latter ornamented with extensive marginal membrane), longer distal spine ornamented with membrane on outer side and plumosities on inner side, plus 5 plumose setae. Leg 3 (Fig. 5A) apron (protopod) bearing small, plumose outer seta and long, plumose inner seta and ornamented with membrane along outer and posterior margins, 4 patches of spinules plus 2 setule-bearing papillae on either side of innermost patch, plus 2 relatively long setules near posterior margin. Endopod 2-segmented, proximal segment small, armed with 1 long plumose seta; segment expanded to form velum fringed with setules along free margin; second segment armed with 6 plumose setae. Exopod 3-segmented, proximal segment small, with pecten-like membrane on inner margin of segment and with an expanded outer distal corner ornamented with strip of membrane located lateral to outer spine plus minute setule on outer margin, outer distal spine slightly curved, not reaching distal border of second segment (Fig. 5a); second segment with inner plumose seta and outer naked spine; third segment with 3 setiform spines increasing in size distally plus 4 inner setae. Leg 4 (Fig. 5B) plumose seta at outer distal corner of protopodal segment; exopod distinctly 2-segmented, first exopodal segment with long outer spine; second segment with 4 spines along oblique outer distal margin; each spine with pecten at base (Fig. 5b).

#### Taxon discussion

The initial description of *C.dussumieri* was provided by Rangnekar (1957). It was later transferred by Pillai (1968) to his new genus *Pseudopetalus* Pillai, 1968, as *Pseudopetalusdussumieri*, but was subsequently transferred to *Sinocaligus* Shen 1957 as *Sinocaligusdussumieri* after *Pseudopetalus* was recognised as a junior synonym of *Sinocaligus* (Boxshall and Montu 1997, Dojiri and Ho 2013). In a recent comprehensive revision by Boxshall and Barton (2023), the complex taxonomic history of *C.dussumieri* was thoroughly examined. They proposed to recognise *Sinocaligus* as a junior subjective synonym of *Caligus* and returned *S.dussumieri* to its original combination as *C.dussumieri*. Boxshall and Barton (2023) also recognised *Caligusrivulatus* Pilla, Vankara and Chikkam, 2012, described from *Lutjanusrivulatus* Cuvier, 1828 in Indian waters, as a junior subjective synonym of *C.dussumieri*. The Malaysian specimen was identified as a mature pre-metamorphic female due to the presence of elongated and slender genital complex and abdomen, which corresponds closely with the developmental stage described in the previous studies by Pilla et al. (2012) and Boxshall and Barton (2023). This particular specimen measured 3.24 mm in total length, a measurement consistent with the total length range of *C.dussumieri*, referred to as *C.rivulatus* by Pilla et al. (2012), ranging from 2.36 to 3.12 mm. Similarly, it exhibited a comparable total length to the Australian specimens, which measured between 3.05 and 3.65 mm.

In comparison to the recent re-description of *C.dussumieri* from Australian waters as provided by Boxshall and Barton (2023), the *C.dussumieri* specimens from Malaysia displayed subtle differences including the presence of a neck-like constriction in the anterior part of the genital complex and the more barrel-shaped genital complex, features also observed in *C.dussumieri* from Indian waters (Rangnekar 1957, Pillai 1967, Pilla et al. 2012). As discussed below, we consider these differences can be explained by the pre-metamorphic stage of development of these sets of specimens.

Another minor difference was noted regarding the postantennal process. In the current study, *C.dussumieri* possessed trisensillate papillae, consistent with the description of Pillai (1985), while the Australian specimens exhibited bisensillate papillae. In addition, a rounded, lobate process was present between the postantennal process and the base of the antenna in the Malaysian female. The female from Malaysia lacked the patch of small setules at the base of the inner protopodal seta of leg 1, present in the other specimens. Furthermore, the tips of spines 2 and 3 on the distal exopodal segment of leg 1 were ornamented with a row of serrations, distinguishing them from previously described specimens from India and Australia.

A pecten-like structure at the outer distal corner of the first exopodal segment of leg 3 was observed in the present specimen, but this feature was not illustrated in previous studies. However, this structure's presence in the Australian material was subsequently confirmed through additional observations of Australian specimens in this study.

## Identification Keys

### Key to species of the *bonito*-group of *Caligus* based on adult females

**Table:** 

1	First abdominal somite grossly enlarged, wider than genital complex	2
–	Abdomen typically slender, rarely enlarged, but never wider than genital complex	4
2	Plumose setae on posterior margin of distal exopodal segment of leg 1 well developed (at least as long as distal spine 3)	*Caligustimorensis* Izawa, 1995
–	Plumose setae on posterior margin of distal exopodal segment of leg 1 vestigial (shorter than distal margin spine 3)	3
3	Abdomen large, peach-shaped, only slightly longer than broad, with deep postero-median lobes	*Caliguscaudatus* Gnanamuthu, 1950
–	Abdomen elongate, tapering anteriorly and posteriorly, approximately 2.4 times longer than wide, lacking postero-median lobes	*Caligusformicoides* Redkar, Rangnekar and Murti, 1949
4	Abdomen enlarged, about as wide as cephalothorax, but slightly narrower than genital complex; sternal furca absent	*Caligusgrandiabdominalis* Yamaguti, 1954
–	Abdomen distinctly narrower than cephalothorax and genital complex; sternal furca present	5
5	Genital complex with well-developed posterolateral lobes, extending to middle of abdomen; first exopodal segment of leg 4 with projecting triangular outer margin	*Caligusinfestans* Heller, 1865
–	Genital complex without posterolateral lobes or with lobes not reaching middle of abdomen; first exopodal segment of leg 4 with linear outer margin	6
6	Abdomen short, about as wide as long and less than half length of genital complex	*Caligusasymmetricus* Kabata, 1965
–	Abdomen distinctly longer than wide (at least 1.5 times) and at least half length of genital complex	7
7	Leg 4 with conspicuous ornamentation of denticles on surface of first exopodal segment	*Caliguspauliani* Nuñes-Ruivo & Fourmanoir, 1956
–	Leg 4 typically with single setule on margin of first exopodal segment, but lacking ornamentation of denticles on surface of segment	8
8	Leg 2 with outer spine on first exopodal segment very elongate with spatulate tip	*Caligusdussumieri* Rangnekar, 1957
–	Leg 2 with outer spine on first exopodal segment typical, tapering towards tip	9
9	Abdomen at most about 2.5 times longer than wide	10
–	Abdomen at least 3 times longer than wide	16
10	Postantennal process reduced (lacking defined tine)	*Caligusphipsoni* Basset-Smith 1898
–	Postantennal process with typical curved tine	11
11	Pair of triangular accessory processes present on body surface either side of sternal furca	*Caligushoplognathi* Yamaguti and Yamasu, 1959
–	Sternal furca lacking associated accessory processes on adjacent body surface	12
12	Genital complex as long as abdomen, with prominent posterolateral lobes	*Caligusbonito* C. B. Wilson, 1905
–	Genital complex at least 1.3 times longer than abdomen, lacking distinct posterolateral lobes	13
13	Abdomen 2-segmented	14
–	Abdomen 1-segmented	15
14	First abdominal somite more than twice as long as small second (anal) somite	*Caligusmalabaricus* Pillai, 1961
–	Both abdominal somites about equal in width and length	*Caliguscossackii* Bassett-Smith, 1898
15	Patches of fine spinules present posteriorly on ventral surface of abdomen	*Caligusmutabilis* Wilson, 1905
–	Ventral surface of abdomen lacking patches of fine spinules posteriorly	*Caligustriabdominalis* Byrnes, 1987
16	Abdomen longer than or about as long as genital complex, distinct indentation between longer and wider anterior part and small posterior region.	*Caligusquadratus* Shiino, 1954
–	Genital complex at least 1.3 times longer than abdomen, no distinct indentation between posterior and anterior regions	17
17	Antenna with small, blunt posterior process on proximal segment	18
–	Antenna with small, pointed posterior process on proximal segment	*Caligusomissus* Cressey and Cressey, 1980
18	Abdomen 1-segmented	19
–	Abdomen 2-segmented, second (anal) somite slightly longer than first abdominal somite	*Caligustenuifurcatus* Wilson, 1937
19	Total body length less than 3 mm; small chitinous process present between antenna and postantennal process	*Caliguschamelensis* Morales-Serna, Pinacho-Pinacho, Gómez and Pérez-Ponce de León, 2014
–	Total body length more than 3 mm; lacking any chitinous process between antenna and postantennal process	*Caligusasperimanus* Pearse, 1951

An identification key distinguishing between species in the *bonito*-group of *Caligus* based on characteristics of adult females is provided following Yamaguti (1954), Shen (1957), Yamaguti and Yamasu (1959), Rangnekar and Murti (1960), Pillai (1961), Cressey and Cressey (1980), Pillai (1985), Byrnes (1987), Cressey (1991), Izawa (1995), Lin and Ho (2001), Ho and Lin (2004), Suarez-Morales et al. (2008), Morales-Serna et al. (2014), Boxshall (2018), Kamanli (2020) and Boxshall and Barton (2023).

## Discussion

In this present study, the genital complex and abdomen of the adult female examined using CLSM displayed differential intensity of staining. It is possible that this differential intensity of staining is an artefact, but we infer that the staining intensity indicates a difference in the properties of the cuticle. The stained specimen is an adult female immediately prior to the post-mating metamorphosis (see Boxshall and Barton (2023)) which involves marked lateral expansion and lengthening of the genital complex and the abdomen resulting in the development of the habitus shown for fully mature *C.dussumieiri* by Pillai (1985). The areas which undergo maximum expansion are the genital complex and anterior abdomen. The posteriormost part of the abdomen, corresponding to the anal somite, does not undergo expansion. It appears that the very lightly stained areas of cuticle correspond to the areas which undergo metamorphic expansion. The cuticle surrounding the genital apertures is heavily stained indicating that it is sufficiently rigid to enable the spermatophores to be deposited and attached securely during mating. It is probable that the area immediately around the genital apertures does not undergo any metamorphic transformation. Similarly, the patch of intensely stained cuticle on the dorsal surface at the rear end of the genital complex is located where the paired maxillipeds of the male might grasp the female during spermatophore transfer. This heavily-stained area might indicate another patch of more rigid cuticle that does not undergo change during the metamorphic expansion of the genital complex. Similar patterns of intensely stained patches were observed in the same regions on the dorsal and ventral surface of the genital complex in 14 other caligid species examined using CLSM and the same staining protocol (Bernot, unpublished), so this appears to be a general pattern and not just an artefact of staining in this specimen of *Caligusdussumieri*.

### Host relationships

*Caligusdussumieri* has been observed to infect two distinct host families, the Lutjanidae and Dussumieriidae. The adult males, copepodid (including chalimus) stages and mated, but not fully metamorphosed adult females were found on *Lutjanusjohnii* from Australian waters (Boxshall and Barton 2023) and *L.rivulatus* from Indian waters (Pilla et al. 2012). Our new Malaysian female at the same stage of development is from *L.malabaricus*. Fully matured females with the typical expanded genital complex and abdomen have been found on the dussumieriids *Dussumieriaacuta* Valenciennes, 1847 and *D.elopsoides* Bleeker, 1849 (as *D.hasseltii* Bleeker) both in Indian waters (Rangnekar 1957, Pillai 1968).

Lutjanids are a group of fish with high commercial value due to their desirable taste, ability to reproduce in captivity and tolerance to a range of salinity regimes which allow them to thrive in various marine environments including estuaries and mangroves as well as the open sea (Ibarra-Castro and Duncan 2007). In the family Lutjanidae, *Lutjanus* is the most species rich genus, currently accommodating 73 species (Froese and Pauly 2023). Twenty-eight species of caligid copepods have been recorded so far from *Lutjanus* species. Amongst these are five species belonging to the *bonito*-group within *Caligus*, namely: *C.asperimanus*, *C.bonito*, *C.irritans*, *C.mutabilis* and *C.tenuifurcatus* (Table 1).

Species of *Caligus*, belonging to the *bonito*-group, are known from a variety of lutjanid hosts around the world. *Caligusasperimanus* and *C.mutabilis* exhibit a broad distribution and host range, having been reported from up to seven different lutjanid hosts in different regions, including Belize (Cressey 1991), the Bahamas, Brazil (Oliveira et al. 2020) and India (Pilla et al. 2012). While about three-quarters of reports of species of the *bonito*-group infecting lutjanids originated from North and South America, four species have been documented as infecting commercially exploited lutjanids from Asia, including *L.johnii* recorded from the coast of India (Pilla et al. 2012), *L.malabaricus* from the Malacca Strait and Terengganu Coast, Malaysia (Leong 1984), *L.argentimaculatus* from the Philippines (Ho et al. 2004) and *L.russellii* from Taiwan (Ho and Lin 2004).

In Malaysia, out of the 20 species of fish previously reported to serve as hosts for caligid copepods, three species of lutjanids are known to collectively host four species of *Caligus*: *Lutjanuserythropterus* hosts *C.chiastos* and *C.torpedinis*; *L.johnii* hosts *C.chiastos*; and *L.malabaricus* hosts *C.malabaricus* (Venmathi Maran et al. 2009).

*Caligusdussumieri* is assigned to the *bonito*-group because of the presence of three plumose setae on the distal exopodal segment of leg 1 and a row of stout denticles along the outer margin of the second endopodal segment of leg 2 (Boxshall and Barton 2023). It also shares two additional characteristics common to members of this species group: a 3-segmented leg 4 with four spines on the second exopodal segment and the presence of a small blunt posterior process on the proximal segment of the antenna. The *bonito*-group is currently composed of 20 species: *C.asperimanus* Pearse, 1951, *C.asymmetricus* Kabata, 1965, *C.bonito* Wilson C.B., 1905, *C.caudatus* Gnanamuthu, 1950, *C.chamelensis* Morales-Serna, Pinacho-Pinacho, Gómez and Pérez-Ponce de León, 2014, *C.cossacki* Bassett-Smith, 1898, *C.dussumieri* Rangnekar, 1957, *C.formicoides* Redkar, Rangnekar & Murti, 1949, *C.grandiabdominalis* Yamaguti, 1954, *C.hoplognathi* Yamaguti and Yamasu, 1959, *C.infestans* Heller, 1865, *C.malabaricus* Pillai, 1961, *C.mutabilis* Wilson, 1905, *C.omissus* Cressey and Cressey 1980, *C.pauliani* Nuñes-Ruivo & Fourmanoir, 1956, *C.phipsoni* Basset-Smith 1898, *C.quadratus* Shiino, 1954, *C.tenuifurcatus* Wilson, 1937, *C.timorensis* Izawa, 1995, and *C.triabdominalis* Byrnes, 1987 (Boxshall 2018, Ohtsuka and Boxshall 2019, Kamanli 2020). *Caligusdussumieri* along with *C.caudatus*, *C.formicoides* and *C.timorensis* were recently added to *bonito*-group after the species were transferred from *Sinocaligus* to *Caligus* (Boxshall and Barton 2023).

Caligids from the *bonito*-group have been recorded from another 30 families of fish, of which eight families (Carangidae, Coryphaenidae, Hemiramphidae, Istiophoridae, Nematistiidae, Pomatomidae, Scombridae and Sphyraenidae) are generally known as pelagic fish and 21 families are demersal fish from families Ariidae, Balistidae, Caesionidae, Centropomidae, Embiotocidae, Ephippidae, Gerreidae, Haemulidae, Kyphosidae, Mugilidae, Mullidae, Oplegnathidae, Polynemidae, Pomacentridae. Rhinobatidae, Sciaenidae, Serranidae, Siganidae and Sparidae. However, they appear to predominantly parasitise members of the family Scombridae. The fact that they primarily infect pelagic fish, but also use many minor demersal fish as hosts may indicate a possible history of evolutionary host-switching from pelagic to demersal fish.

An interesting example of host-switching during development is known in *C.pauliani* (previously reported as *C.biseriodentatus*), which infects different hosts at different life stages. The adult females are known from the frigate tuna *Auxisthazard* Lacepède, 1800, while premetamorphic adults and copepodid stages characterised mainly by smaller size, slender genital somites and not bearing eggs, are known from a variety of host species belonging to the genus *Scomberomorus* Lacepède, 1801. This form of host-switching appears to involve mated females only given that both pre-metamorphic adult females and males occur on the first host, which is where mating takes place, while only the mated females are known from the second host where the metamorphic developmental changes are completed. Cressey and Cressey (1980) speculated that host-switching might have resulted from predatory activities of the host because *A.thazard* is known as a common and important prey item for many larger fishes (Frimodt 1995).

**Figure 6.**
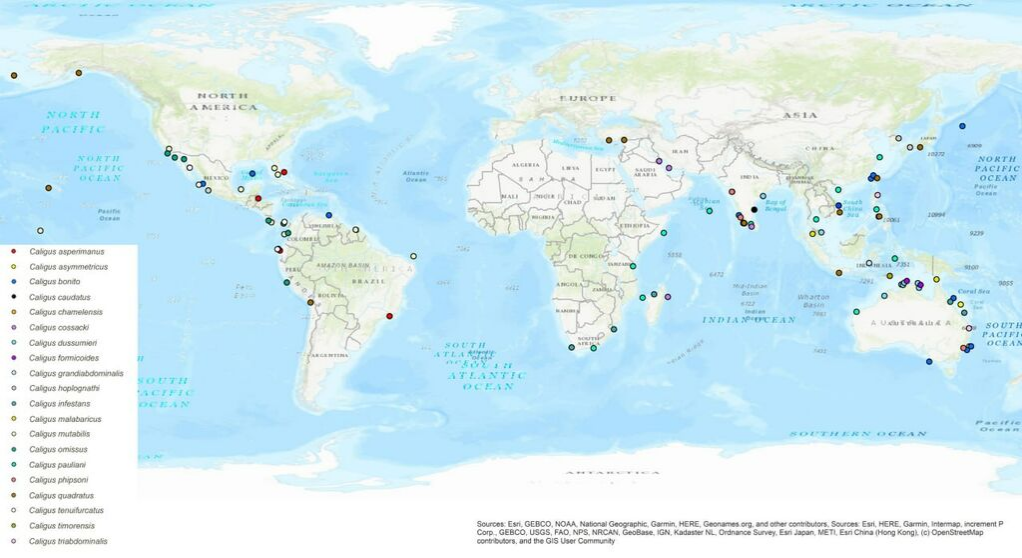
Geographical distribution of species belonging to the *bonito*-group within *Caligus*.

The *bonito*-group has a broad distribution in low to middle latitudes across all oceans, with a denser concentration in the Indo-Pacific. The distribution of species infecting Lutjanidae is primarily concentrated in low latitudes due to the abundance of lutjanid fish in tropical and subtropical areas, as indicated in Fig. 6. In Malaysia, two caligids belonging to the *bonito*-group (*C.dussumieri* and *C.malabaricus*) have been documented in association with two lutjanid fish species (present study and Leong (1984)). Notably, the exploration of caligid copepod diversity in neighbouring Southeast Asian countries has been limited, reflecting a broader scarcity of research on Caligidae in the region. In addition to Malaysia records, a total of five caligids species from the *bonito*-group have been identified from Southeast Asian countries. These include *C.bonito* from *E.affinis* and *C.formicoides* from *D.elopsoides* from *Vietnam* (Truong et al. 2022,) *C.grandiabdominalis* from *Caesiocuning* Bloch, 1791 in Indonesia (Yamaguti 1954), *C.pauliani* recorded from *Scomberomorus* spp. from Thailand, the Philippines and Indonesia (Cressey and Cressey 1980); and *C.quadratus* on Siganidae and Mullidae in the Philippines and Indonesia (Ho et al. 2004, Yuniar et al. 2007). This underscores the need for further investigations to improve our understanding of caligid copepod diversity and distribution in Southeast Asian waters.

## Supplementary Material

XML Treatment for Caligus dussumieri

## Figures and Tables

**Table 1.** Caligid copepods recorded from the genus *Lutjanus* Bloch,1790.

**Lutjanus fish host**	***Caligus* species**	**Geographic distribution**	**References**
*Lutjanusanalis* (Cuvier, 1828)	*Caligusasperimanus* Pearse, 1951	Carrie Bow Cay, Belize	Cressey (1991)
	*Caligusasperimanus* Pearse, 1951	Bahamas	Cressey (1991)
	*Caligusasperimanus* Pearse, 1951	off the coast of Rio de Janeiro, Brazil.	Oliveira et al. (2020)
	*Caligusrobustus* Bassett-Smith, 1898	Mexico	Cressey (1991)
*Lutjanusapodus* Walbaum, 1792	*Caligusasperimanus* Pearse, 1956	Carrie Bow Cay, Belize	Cressey (1991)
	*Caligusatromaculatus* Wilson, 1913	Venezuela	Lagarde (1991)
	*Caligustenax* Heller, 1865	Venezuela	Lagarde (1991)
	*Caligusrobustus* Bassett-Smith, 1898	Belize	Cressey (1991)
	*Caligusxystercus* Cressey, 1991	Belize	Cressey (1991)
*Lutjanusargentimaculatus* Forsskål, 1775	*Caligusepidemicus* Hewitt, 1972	Philipines	Ho et al. (2004)
	*Caliguslutjani* Ho, Lin and Chang, 2007	Taiwan	Ho et al. (2007)
	*Caligusquadratus* Shiino, 1954	Taiwan	Ho et al. (2007)
*Lutjanusargentiventris* Peters, 1869	*Caligusasperimanus* Pearse, 1961	Manabí Coast, Ecuador	Morales-Serna et al. (2016)
	*Caligusdiaphanus* Nordmann, 1833	Jaramijó, Ecuador	Morales-Serna et al. (2015)
	*Caliguslatigenitalis* Shiino, 1954	Bahía de Chamela, Mexican Pacific	Morales-Serna et al. (2014)
	*Caliguslatigenitalis* Shiino, 1955	Jaramijó, Ecuador	Morales-Serna et al. (2015)
	*Caligusserratus* Shiino, 1965	Mexican Pacific	Morales-Serna et al. (2012), Morales-Serna et al. (2014)
	*Caligusschistonyx* Wilson, 1905	Jaramijó, Ecuador	Morales-Serna et al. (2015)
	*Caligustenuifurcatus* Wilson, 1937	Mexican Pacific	Suarez-Morales et al. (2008), Morales-Serna et al. (2012), Morales-Serna et al. (2015)
*Lutjanusbohar* Forsskål, 1775	*Caliguslutjani*	Taiwan	Ho et al. (2007)
*Lutjanuscolorado* Jordan and Gilbert, 1882	*Caligussclerotinosus* Roubal, Armitage and Rohde, 1983	Bahía de Chamela,Mexican Pacific	Morales-Serna et al. (2014)
*Lutjanuserythropterus* Bloch, 1790	*Caliguschiastos*	Malaysia	Muhd-Faizul et al. (2012)
	*Caligusclemensi*	Penang, Malaysia	Yahaya et al. (2022)
	*Caligustorpedinis* (as *C.rotundigenitalis*)	Malaysia	Venmathi Maran et al. (2009)
*Lutjanusfulviflamma* Forsskål, 1775	*Caliguschiastos*	Taiwan	Ho and Lin (2004)
	*Caliguschiastos*	Moreton Bay, Australia	Boxshall (2018)
	*Caligusepidemicus*	Moreton Bay, Australia	Boxshall (2018)
*Lutjanusfulvus* Forster, 1801	*Caligusmutabilis*	Palmyra Atoll, Central Pacific	Soler-Jimenez et al. (2019)
*Lutjanusgriseus* Linnaeus, 1758	*Caligusatromaculatus* Wilson, 1913	Florida	Cressey and Nutter (1987)
	*Caligusbonito*	Isla de Margarita,Venezuela	Fuentes Zambrano et al. (2003)
	*Caligusbonito*	Gulf of Mexico	Ho and Lin (2004)
	*Caligusirritans* Heller, 1869	Venezuela	Lagarde (1991)
	*Caliguswilsoni* Delamare Deboutteville and Nunes Ruivo, 1958	Charlotte Harbor, Florida	Cressey (1991)
*Lutjanusguttatus* Steindachner, 1869	*Caligusdiaphanus* Nordmann, 1834	Jaramijó, Ecuador	Morales-Serna et al. (2016)
	*Caligusmutabilis*	Mexican Pacific	Morales-Serna et al. (2012), Morales-Serna et al. (2014)
	*Caligussclerotinosus*	Bahía de Chamela,Mexican Pacific	Morales-Serna et al. (2015)
*Lutjanusjocu* Bloch and Schneider, 1801	*Caligusasperimanus*	Carrie Bow Cay, Belize	Cressey (1991)
	*Caligusasperimanus*	off the coast of Rio de Janeiro, Brazil.	Oliveira et al. (2020)
*Lutjanusjohnii* Bloch, 1792	*Caligusasperimanus*	India Coast	Pilla et al. (2012)
	*Caligusauriilus* Boxhall and Barton, 2023	Australia	Boxshall and Barton (2023)
	*Caliguschiastos*	Malaysia	Venmathi Maran et al. (2009)
	*Caligusdussumieri*	Australia	Boxshall and Barton (2023)
*Lutjanusmonostigma* Cuvier, 1828	*Caligusmutabilis*	Palmyra Atoll, Central Pacific	Soler-Jimenez et al. (2019)
*Lutjanusmalabaricus*	*Caligusmalabaricus*	Malacca Strait, Malaysia	Leong (1984)
	*Caligustorpedinis* (as *C.rotundigenitalis*)	India	Pillai (1967)
*Lutjanusnovemfasciatus* Gill, 1862	*Caligusbonito*	Mexican Pacific	Ho and Lin (2004), Morales-Serna et al. (2012)
*Lutjanusperu* Nichols and Murphy, 1922	*Caligusmutabilis*	Mexican Pacific	Morales-Serna et al. (2012), Morales-Serna et al. (2015)
*Lutjanusperu* Nichols & Murphy, 1922	*Caligusdiaphanus*	Bahía de Chamela,Mexican Pacific	Morales-Serna et al. (2014)
	*Caligussclerotinosus*	Bahía de Chamela,Mexican Pacific	Morales-Serna et al. (2014)
*Lutjanusrivulatus* Cuvier, 1828	*Caligusnengai* Rangnekar, Rangnekar & Murti, 1953	Taiwan	Ho and Lin (2004)
	*Caligusdussumieri*	India	Pilla et al. (2012)
*Lutjanusrussellii* Bleeker, 1849	*Caligusquadratus*	Taiwan	Ho and Lin (2004)
	*Caligustorpedinis* (as *C.rotundigenitalis*)	Taiwan	Ho and Lin (2004)
	*Caliguspagrosomi* Yamaguti, 1939	Taiwan	Ho and Lin (2004)
	*Caliguslaticaudus*	Taiwan	Ho and Lin (2004)
*Lutjanussynagris* Linnaeus, 1758	*Caligusasperimanus*	off the coast of Rio de Janeiro, Brazil.	Oliveira et al. (2020)
	*Caligusasperimanus*	Carrie Bow Cay, Belize	Cressey (1991)
	*Caligusatromaculatus*	Venezuela	Lagarde (1991)
	*Caliguspraetextus* Bere, 1936	Charlotte Harbor, Florida	Cressey (1991)
	*Caligusrufimaculatus* Wilson, 1905	Florida and the Gulf of Mexico	Cressey (1991)
	*Caliguspraetextus*	Belize; Florida	Bere (1936), Cressey (1991)
*Lutjanusvitta* Quoy and Gaimard, 1824	*Caliguslaticaudus*	Taiwan	Ho and Lin (2004)
	*Caligustorpedinis* (as *C.rotundigenitalis*)	Taiwan	Ho and Lin (2004)
*Lutjanusvivanus* Cuvier, 1828	*Caligusasperimanus*	off the coast of Rio de Janeiro, Brazil.	Oliveira et al. (2020)
*Lutjanus* spp.	*Caligusproductus* Dana, 1852	Mexican Pacific	Morales-Serna et al. (2012)
